# Development and Application of Surface-Enhanced Raman Scattering (SERS)

**DOI:** 10.3390/nano14171417

**Published:** 2024-08-29

**Authors:** Zhenkai Huang, Jianping Peng, Liguo Xu, Peijiang Liu

**Affiliations:** 1 School of Materials and Energy, Foshan University, Foshan 528000, China; hzk@fosu.edu.cn; 2 School of Environmental and Chemical Engineering, Foshan University, Foshan 528000, China; pjp@fosu.edu.cn; 3 College of Light Chemical Industry and Materials Engineering, Shunde Polytechnic, Foshan 528333, China; 21099@sdpt.edu.cn; 4 Reliability Physics and Application Technology of Electronic Component Key Laboratory, The 5th Electronics Research Institute of the Ministry of Industry and Information Technology, Guangzhou 510610, China

**Keywords:** SERS, nanostructures, detection, applications

## Abstract

Since the discovery of the phenomenon of surface-enhanced Raman scattering (SERS), it has gradually become an important tool for the analysis of material compositions and structures. The applications of SERS have been expanded from the fields of environmental and materials science to biomedicine due to the extremely high sensitivity and non-destructiveness of SERS-based analytical technology that even allows single-molecule detection. This article provides a comprehensive overview of the surface-enhanced Raman scattering (SERS) phenomenon. The content is divided into several main sections: basic principles and the significance of Raman spectroscopy; historical advancements and technological progress in SERS; and various practical applications across different fields. We also discuss how electromagnetic fields contribute to the SERS effect, the role of chemical interactions in enhancing Raman signals, a modeling and computational approaches to understand and predict SERS effects.

## 1. Introduction of Raman Scattering Spectroscopy

**Figure 1:**
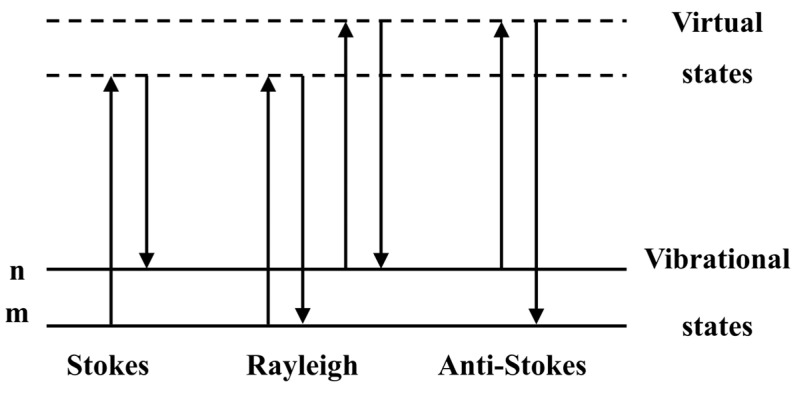
Illustration of Rayleigh and Raman scattering.

In 1923, a German physicist, Smekal, proposed the existence of intrinsic inelastic scattering of light [1]. In 1928, two Indian physicists, Raman and Krishnan, using mercury lamps, prisms, and photographic substrates as experimental tools, discovered the phenomenon of inelastic scattering of liquid benzene for the first time. The inelastic scattering produced by the interaction of a vibrating molecule with incident light was named Raman scattering [2]. Raman scattering is a scattering phenomenon that can be utilized to detect molecular vibrations [3,4]. As shown in Figure 1, when a sample is irradiated by a monochromatic laser beam, the sample molecules interact with the laser beam and emit scattered light in all directions. The frequency of most of the incident light and its scattered light is the same, which is called Rayleigh scattering, while a portion of the scattered light differs from the incident light frequency, which is called Raman scattering. In this case, Stokes lines appear when the frequency of the scattered radiation is lower than the frequency of the incident light. Alternatively, anti-Stokes lines appear when the frequency of the scattered radiation is higher than the frequency of the incident radiation [5,6]. In Raman scattering, the momentum of photons changes while the photons exchange energy with molecules. Therefore, parameters such as the intensity, number, and displacement of peaks in the Raman spectrum contain information about the structure and composition of the excited molecules. Raman scattering is prevalent in a wide variety of substances, including gases, liquids and solids. Each different substance has its own characteristic “fingerprint” Raman spectrum, enabling different substances to be identified and characterized through Raman spectroscopy.

Since its discovery, Raman spectroscopy has quickly emerged as a potent analytical technique for detecting chemical compositions and other important information [7]. For instance, Raman spectroscopy possesses notable advantages over infrared spectroscopy, one of which is its lower susceptibility to the test environment, including ambient carbon dioxide and water. Consequently, Raman spectroscopy has a wide range of applications. However, it is not a highly sensitive technique due to the weak Raman scattering effect, which significantly limits the practical application of Raman spectroscopy [8]. In the early stages of Raman scattering technology, a mercury lamp was utilized as a novel excitation light source. Until 1960, lasers were developed as the excitation light source for Raman scattering. Compared to the traditional mercury lamp, lasers exhibit characteristics such as high power, high energy, excellent monochromaticity, and polarizability, which have facilitated the rapid development of the Raman scattering technique and led to its widespread use in various domains [9].

## 2. Development of SERS Technology

Although Raman scattering techniques possess numerous advantages, the Raman scattering signal is so weak that it is difficult to detect using conventional laser Raman spectrometers. It was not until the 1970s that Fleischmann et al. [10], at the University of Southampton in the UK, accidentally discovered the ultra-intense Raman spectrum of pyridine on the surface of a rough silver electrode. Subsequently, Jeanmaire, Van Duyne et al. [11] and Albrecht et al. [12] also discovered and verified the existence of this phenomenon. To explain this phenomenon, they suggested that the enhancement of the ultra-strong Raman signal is not only due to the increase in the number of adsorbed molecules on the surface, but also due to the existence of some kind of Raman scattering enhancement effect of the adsorbed molecules on the substrate surface. However, this work on Raman surface enhancement effects underwent a lengthy review process before it was published. Shortly thereafter, the American Chemical Society took notice to this phenomenon and explained it as a resonance-like Raman effect, i.e., the surface-enhanced Raman scattering (SERS) effect. Specifically, it is generally agreed that SERS results from the amplification of electromagnetic fields generated by the excitation of localized surface plasmons [13,14]. In particular, when probe molecules are within the radiation range of the nanoparticles, the Raman scattering of probe molecules can even be enhanced by tens of billions of times, which completely compensates for the shortcomings of the low sensitivity of Raman scattering spectroscopy, making the SERS technique a promising candidate for development in the areas of single-molecule detection and probing applications. Therefore, the mechanism of SERS has become an exciting scientific phenomenon and has gradually become a research hotspot in related fields.

## 3. Theoretical Mechanisms for the Emergence of the SERS Phenomenon

The study of the mechanism of the SERS phenomenon is important for the development of SERS techniques. However, the accurate results of this study are still inconclusive. Theoretical simulations have been utilized to study the mechanism of the SERS phenomenon, and the theoretical modelling of SERS has a long history and has undergone many modifications [15]. After a large number of experimental verifications, researchers in the field of SERS have reached a consensus that the Raman enhancement factor is caused by the electromagnetic enhancement effect of plasma excitation of electrons in metal particles and the chemical enhancement effect of the electron transfer of the probe molecules in metal particles. The electromagnetic enhancement effect is considered to be the main factor in the generation of SERS. The electromagnetic enhancement factors are mainly related to the following points:

(1) Relating to the material of the SERS substrate: the SERS substrates refer to those materials that could provide the plasma resonance to produce the SERS effect. These materials generally refer to metallic materials such as gold, silver, copper, and aluminum, as well as other materials that are being investigated, such as electrolytes and semiconductor materials.
(2) Related to “hot spots”: the electromagnetic enhancement on the surface of the SERS substrate is usually not uniform. Typically, the enhancement mainly occurs in very small and narrow areas, i.e., “hot spots”. From the physical structure, the signal enhancement is more efficient at the sharp tips and the gaps between the nanoparticles.
(3) Distance-dependent: as the distance between the nanoparticles and the surface increases, the Raman signal intensity decreases very rapidly since the probe molecules need to be kept within 10 nm of the substrate surface for better enhancement.

The following section explains the reasons for the emergence of SERS in terms of various mechanisms.

### 3.1. Electromagnetic Enhancement Mechanism

**Figure 2:**
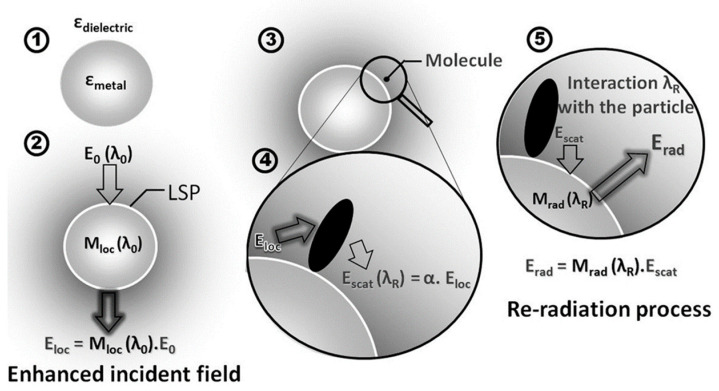
The schematic diagram of SERS electromagnetic enhancement. (1) A metallic nanoparticle with permittivity ε_metal_ surrounded by a dielectric medium with permittivity ε_dielectric_. (2) Nanoparticle excitation by an incident field E_0_ at a wavelength λ_0_ creating a local field E_loc_ proportional to E_0_ and to the enhancement factor M_loc_(λ_0_). (3) Molecule at the vicinity of the nanoparticle. (4) The local field polarizes the molecule which has a scattered field E_scat_ at the Raman wavelength λ_R_ and proportional to the molecule polarizability α. (5) The field scattered by the molecule interacts with the nanoparticle creating a re-radiated field E_rad_ proportional to Escat and to the enhancement factor M_rad_(λ_R_) [18]. Copyright © 2012 Elsevier Ltd.

The electromagnetic enhancement effect is generally produced on the surface of special nanostructures. The effect is caused by the collective vibration of conduction electrons in metallic nanoparticles. The vibration of the conduction electrons leads to the enhancement of the localized electromagnetic field, which greatly enhances the intensity of the Raman scattering of molecules within the range [16,17]. The principle of the electromagnetic enhancement effect is illustrated in Figure 2 [18]. The interaction between the incident photon beam with wavelength λ_0_ and the nanostructures results in the localized enhancement of the incident field E_0_. Molecules within the radiation range of the nanoparticles also experience the enhanced electric field strength E_LOS_, where E_LOS_ = M_loc_(λ_0_)E_0_ (M_loc_(λ_0_) is the enhancement factor of the localized electric field). Upon excitation by the enhanced electromagnetic field, these molecules scatter a Raman signal at a wavelength of λ_R_ (λ_R_ ≠ λ_0_). Moreover, the scattered field E_scat_ radiated by photons at a wavelength of λ_R_ is also enhanced compared to the scattered field of the molecule in the absence of the nanostructure present (E_scat_ = αE_LOS_ = αM_loc_(λ_0_)E_0_). The interaction between the scattered field E_scat_ and the nanoparticle causes it to be enhanced again. As a result, the field is radiatively enhanced again as E_SERS_ = M_rad_(λ_R_)E_scat_ = M_rad_(λ_0_)αE_LOC_ = αM_loc_ (λ_0_)M_rad_ (λ_R_)E_0_, so that the I_SERS_ is proportional to the |E_SERS_|^2^ for the Raman scattering intensity of the molecule within the radiation range of the nanoparticles. Since the field enhancement at a particular incident wavelength can occur twice consecutively, the molecule is finally subjected to a total electric field enhancement of |E_SERS_|^4^, making the SERS effect significant.

However, there are also some limitations to electromagnetic enhancement, mainly because its enhancement decreases rapidly with increasing distance between the probe molecules and the metal nanoparticles. The range of the effect is generally from 10 to 100 Å, which is considered a long-range enhancement effect and makes SERS a surface-sensitive technique [19,20].

**Figure 3:**
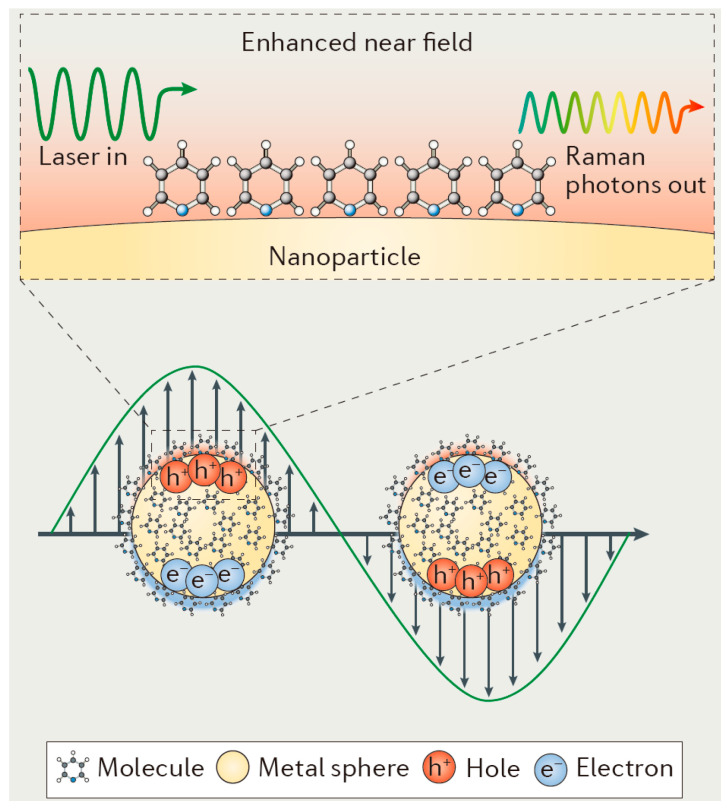
Schematic of plasmon oscillation for a metal nanosphere [21]. Copyright © 2016, Macmillan Publishers Limited.

Surface plasmon resonance (SPR) causes molecules within the radiation range of nanoparticles to experience an enhancement of the local electric field [21]. Although the increase in the local electric field is generally insignificant, the intensity of inelastically scattered light is enhanced to the fourth power of the original intensity, resulting in a very significant SERS effect (Figure 3). When free electrons on the surface of a metallic nanoparticle are excited by electromagnetic wave irradiation and undergo a collective oscillation phenomenon which generates an electromagnetic wave, and when the frequency of the incident electromagnetic wave is the same as the intrinsic frequency of the oscillating free electrons, the SPR phenomenon occurs [22]. The localized surface plasma resonance (LSPR) refers to the SPR phenomenon confined to a small range of the metal surface. Generally, the surface of some noble metals can produce the SERS effect with an enhancement factor of 10^6^~10^11^. The SPR phenomenon is reflected in the spectral characteristics of the noble metal nanoparticles, mainly in the absorption bands generated in the visible range. The incident laser frequency determines the intensity of the plasma resonance generated on the surface of the noble metal nanoparticles. When the Raman scattering frequency slightly deviates from the frequency of the incident light and both the Raman scattering and the incident laser frequency are located near the plasma resonance frequency, the resonance occurs and produces an enhancement of the |E_SERS_|^4^. When the Raman scattering frequency deviates significantly from the incident laser frequency, the incident light and the Raman scattering cannot be simultaneously resonated with the plasma, and the electrostatic force is increased. Moreover, when the Raman scattering frequency deviates greatly from the incident laser frequency, the incident light and Raman signal cannot resonate with the plasmon at the same time, so the electric field enhancement effect is sharply reduced. In addition, when Raman scattering occurs, the plasmon oscillation direction must be perpendicular to the surface of the substrate, so it is generally necessary to use a curved or rough surface to generate localized collective oscillations. Generally, nanoparticles with a size of 100 nm or less are a very good choice as the SERS substrates [23].

The noble metals Au, Ag, and Cu are the most prevalent SERS substrates. While their corresponding LSPR peaks all reside in the visible region, these peaks exhibit distinct characteristics, with respective peak positions at 2.1 eV, 2.3 eV, and 3.5 eV [24]. Notably, the positions of the LSPR peaks for the same noble metal SERS substrates are also not entirely fixed. This variability arises because the location of the LSPR peaks is influenced not only by the metal type but also by factors such as the metal’s structure, size, shape, aggregation state, and dielectric environment [25]. Consequently, the LSPR peaks of noble metals are typically modulated by adjusting various parameters, including the ratio between components [26]. As a result, it is common practice to adjust the position of the LSPR peaks of noble metals to the proximity of a specific incident wavelength, aiming to maximize the enhancement of the electromagnetic field and achieve the strongest SERS effect.

In addition, “hot spots” represent special regions of strong spatial localization, characterized by significantly enhanced local electric fields induced by LSPR [27]. The “hot spots” effect typically manifests at connections or gaps between noble metal nanoparticles, or in highly curved regions with a small radius of curvature, such as the corners or tips of nanostructures (the “lightning rod” effect), or at topographies with nanometer-scale roughness, etc. [28,29,30]. Therefore, SERS enhancement factors of up to 10^15^ at “hot spots” enable single-molecule detection [31,32]. However, there is a pronounced spatial limitation at the “hotspot”, where only the Raman scattering of molecules located within the “hot spots” region on the surface of the noble metal substrate undergoes substantial enhancement [33]. The Raman scattering of molecules situated outside the “hot spots” range remains largely unaffected. Owing to the uneven spatial distribution of the “hot spots”, the Raman scattering typically varies with the location. Importantly, it has been found that the probe molecules (constituting less than 0.01% of the total number of molecules) residing within the “hot spots” range exhibit an enhancement factor exceeding 10^9^, contributing to a quarter of the total Raman signal intensity [34]. The highly random dispersion of “hot spots” poses a challenge to the quantitative detection of SERS, rendering the enhancement factor and Raman scattering intensity of the noble metal substrate unrepeatable for each detection. Therefore, the development of a SERS substrate with a uniform distribution of “hot spots” is necessary for the quantitative detection of SERS.

### 3.2. Chemical Enhancement Mechanism

While the electromagnetic enhancement mechanism can adequately explain most SERS phenomena occurring on the surface of noble metal substrates, there are still exceptions [35]. Despite the known non-selectivity of the electromagnetic enhancement mechanism, the Raman scattering enhancement of CO and N_2_ molecules within the radiation range of the surface exhibits significant variation [36]. Specifically, the SERS enhancement factor for these two molecules, which possess similar Raman scattering properties, differs by a factor of 200 under the same experimental conditions. Subsequent studies revealed that the presence of chemisorption between the molecules and the noble metal substrate, or the ability of molecules to interact with the substrate active site, are highly correlated with the strength of its Raman scattering. These findings suggest that there are still other unknown factors that contribute to the enhancement of the Raman scattering, alongside electromagnetic enhancement.

This Raman scattering enhancement generated by the interaction between the substrate and the probe molecule is termed chemical enhancement, and the chemical enhancement mechanism likely comprises the following three components [37]:

(1) Resonance enhancement of the probe molecule;
(2) Charge transfer resonance enhancement between the probe molecule and the substrate;
(3) Non-resonance alteration enhancement, which refers to the static chemical enhancement of the molecular polarization rate due to interaction.

Among them, the resonance enhancement between the incident light and the probe molecule is independent of the substrate and is generally categorized into the class of resonance Raman spectroscopy. The SERS enhancement factor generated by the resonance between the incident light and the probe molecule usually reaches orders of magnitude of 10^3^ to 10^4^. In addition, the Raman scattering of probe molecules adsorbed on the substrate surface differs from the Raman scattering of free molecules without interaction. When probe molecules chemically interact with the substrate surface, the interaction between the probe molecules and the substrate or other substances adsorbed on the substrate surface can alter the scattering cross-sectional area of the probe molecules in the Raman vibrational modes as well as their molecular polarizabilities, resulting in the enhancement of the Raman scattering [38]. This is the role of chemical enhancement [39,40,41].

The chemical enhancement effect is now recognized as an independent mechanism that could enhance the Raman scattering of probe molecules interacting with metal surfaces [42]. The fundamental aspects of chemical enhancement depend on the dependence of the analyte molecule or structure on chemical properties. Furthermore, while electromagnetic enhancement effects are non-selective for chemical properties, chemical enhancement effects are entirely dependent on the chemical properties of the molecule itself. For example, differences in enhancement effects of two or more orders of magnitude due to chemical enhancement effects can be observed between the CO and N_2_ molecules [36]. In most cases, both electromagnetic and chemical enhancement effects coexist, where electromagnetic enhancement can achieve higher enhancement effects, while chemical enhancement is usually considered to be less effective than electromagnetic enhancement [43]. Although there is a consensus on the existence of chemical enhancement, the underlying enhancement mechanism remains unclear. For instance, experimental observations and theoretical estimates in recent years have suggested that the enhancement factor of chemical enhancement may be as high as 10^5^ to 10^7^ [44].

### 3.3. Theoretical Modeling of SERS Enhancement Factors

Over the past decade, with the accumulation of numerous experiments and the continuous advancement of numerical processing methods, alongside the development of computer technology, a theoretical and technical foundation has been laid for the systematic investigation of the relationship between metal nanoparticles and their properties. For the simulation of the SERS effect, various numerical processing methods have been proposed, such as solving Maxwell’s equations, to quantitatively calculate the electromagnetic enhancement factor. Currently, there exist several numerical approaches for modeling the interaction between material systems and electromagnetic waves, including the finite difference time domain (FDTD) method [45,46,47,48,49,50], the pseudospectral time domain (PSTD) method [51,52], the T-array method [53,54], the finite element method (FEM), the boundary element method (BEM) [55,56,57,58,59], and the discrete dipole approximation (DDA) [60,61]. These numerical methods have played an increasingly important role in the fields of electromagnetic wave propagation and scattering, spectroscopy, near-field technology, and sensors. Among them, the FDTD method has been successfully utilized to calculate and simulate the SERS effect [62,63,64].

In 1966, Yee et al. [45] first proposed the FDTD numerical algorithm to address the issue of electromagnetic pulse propagation and reflection within electromagnetic media by directly differentiating Maxwell’s equations. Initially, the electromagnetic field is constituted by the alternating propagation of variable magnetic and electric fields. Therefore, the value of the electric field at a specific spatial point at a given time depends on its value at the preceding time step at that point and the distribution of the magnetic field around it, and vice versa for the magnetic field. For instance, four magnetic field components surround one magnetic field component. This spatial sampling of electromagnetic field components not only aligns with the natural structure of Ampere’s loop law and Faraday’s law of electromagnetic induction but also conforms to the differential calculation of Maxwell’s equations. Consequently, given the appropriate initial conditions of the electromagnetic problem, the electric and magnetic field strengths can be determined step by step through spatial alternation, providing the spatial electromagnetic field distribution at each instant. The fundamental principle of the FDTD numerical algorithm is to compute the spatial nodes in the time domain using the Yee metric method, where the nodes of the magnetic and electric fields in the electromagnetic field are discretized by alternating sampling in both time and space. This approach discretizes the time domain of Maxwell’s equations, transforming them into explicit difference equations, thereby significantly simplifying the computational process. Furthermore, the FDTD algorithm incorporates the computational method of absorbing boundary conditions, which allows the computation to be performed within a limited spatial range, reducing the complexity of the computational procedure and mitigating the demand on computer hardware.

Metallic materials are dispersive, with their dielectric coefficient varying as a function of frequency. It was not until the 1990s that the FDTD method commenced its application to this particular type of material [47]. The material properties of metals are generally characterized as modified Debye materials, exhibiting distinct dielectric constants. In conducting FDTD calculations, to ensure the reliability of the material parameters, all parametric calculations necessitate direct fitting using experimentally determined values of the material dielectric coefficients.

### 3.4. Evaluation of SERS Enhancement Factor

The limit of detection and the Raman enhancement factor are two crucial parameters for evaluating the Raman scattering of SERS substrates. However, the calculated value of the enhancement factor is influenced by the Raman detection method. Moreover, variations in SERS substrate materials, excitation wavelengths, and probe molecule species could also impact the calculated results of Raman scattering [65]. Additionally, the estimation method employed in the actual calculation process affects the obtained enhancement factor values. Consequently, depending on different test methods, test conditions and estimation methods, the enhancement factor can be defined in various ways, including the single-molecule enhancement factor, the SERS substrate enhancement factor, and the analytical enhancement factor [66]. The single-molecule enhancement factor is generally used to calculate the Raman scattering of specific molecules. It is quite challenging to calculate and obtain the single-molecule enhancement factor due to the precise definition of a single molecule. For instance, Wang et al. presented a single-molecule spectral system based on a gold plasmonic nanopore for analyzing two peptides and their single-point mutated variants. The gold plasmonic nanopore enabled the high-throughput acquisition of SERS spectra at the single-molecule level by electrically driving analytes into “hot spots” [67]. Generally, researchers are more interested in calculating the average enhancement factor produced by molecules rather than calculating the exact Raman signal intensity of each molecule distributed on the surface of the SERS substrate. Therefore, the SERS substrate enhancement factor is commonly used to calculate the average Raman enhancement factor of the SERS substrate, employing the general formula:

(1) $$Enhancement\;factor=\frac{I_{surf}/N_{surf}}{I_{bulk}/N_{bulk}}$$

In this context, *I_surf_* and *I_bulk_* represent the intensities of the same characteristic peaks in SERS and normal Raman detection, respectively. Similarly, *N_surf_* and *N_bulk_* denote the effective number of molecules that can be detected within the Raman laser irradiation area, respectively. If the SERS substrate surface is assumed to be adsorbed as a single molecular layer, the calculation of *N_surf_* can be approximated as follows:

(2) $$N_{surf}=\frac{RA}{\sigma }$$

In this equation, *R* represents the roughness factor of the SERS substrate, *A* denotes the spot area within the laser irradiation region, and *σ* signifies the surface area occupied by molecules on the surface of the SERS substrate. The calculation of *N_bulk_* is based on the confocal characteristics of the system, as the collection efficiency of the scattered photons of the molecules being measured varies with the change in confocal depth. By utilizing the data of the specific surface area of the substrate and the number of molecules adsorbed on its surface, and assuming that the molecules form a complete monolayer, *N_surf_* and *N_bulk_* can be calculated using the following equation:

(3) $$N=C\cdot N_{A}\cdot Sh$$

In this equation, *C* denotes the concentration of the probe molecule, *N_A_* represents Avogadro’s constant, *h* is the test focusing depth, and *S* signifies the specific surface area of the SERS substrate *S_surf_*, which in conventional Raman measurements corresponds to the laser spot area, *S_bulk_*. By integrating the above three formulas, the enhancement factor of the SERS substrate can be calculated as follows:

(4) $$Enhancement\;factor=\frac{I_{surf}C_{bulk}S_{bulk}}{I_{bulk}C_{surf}S_{surf}}$$

Currently, the method of assessing the basal SERS effect using calculated enhancement factor values remains controversial, owing to the persistent differences between the single-molecule enhancement factor and the SERS substrate enhancement factor. Objectively speaking, the calculation of single-molecule enhancement factors is considered more accurate and reasonable, while the calculation of SERS substrate enhancement factors is deemed more practical. Due to the significant spatial localization of the “hot spots”, the single-molecule enhancement factor exhibits considerable variation with position. Therefore, the single-molecule enhancement factor is generally utilized to precisely analyze the contribution of a single molecule to the Raman enhancement, whereas the SERS substrate enhancement factor is more practical for analytical purposes. Furthermore, the SERS substrate enhancement factor is usually lower than the maximum single-molecule enhancement factor. In addition, when applying the SERS substrate enhancement factor in practical calculations, it is often challenging to obtain the parameter data precisely. Hence, reasonable estimation assumptions are frequently made based on the actual test conditions. As a result, the values of SERS substrate enhancement factors calculated by different researchers can exhibit significant variation due to differences in their respective estimation assumptions or descriptions. Therefore, the development of a more unified standard is imperative for measuring the strength of the SERS enhancement factor of the substrate.

## 4. Application of SERS Technology

SERS-based detection is a highly efficient and non-destructive trace analysis technique capable of both single and composite molecules. Compared with traditional analytical methods, SERS can accurately identify chemical substances and simultaneously analyze their structures [68], attributed to the following characteristics: (1) ultra-high sensitivity, enabling detection at the single-molecule level; (2) spectral information that serves as a “fingerprint” of molecule identification; (3) ultra-narrow signal peak bandwidth [69,70]; (4) good stability exhibiting resistance to photodegradation and bleaching; (5) multiple detection functions; and (6) an adjustable substrate structure that can be tuned for detecting different substances. To date, the application of SERS has expanded from the fields of materials and environmental sciences to biomedicine, encompassing physical, chemical, and analytical techniques. In recent years, one of the main driving forces behind the progress in SERS research has been the tremendous advancement in nanoparticle preparation technology. SERS substrates are usually composed of nanostructures, and the structure of the nanoparticles can be precisely tuned during the synthesis process, including their size, morphology, and inter-particle gaps, which renders the preparation of SERS substrates highly controllable. Based on the precise and controlled preparation of nanoparticles, SERS substrates can be produced quickly, economically, reproducibly, and on a large scale for a wide range of applications, such as pollutant detection, reactive process detection, biosensing, archaeology, and art analysis.

### 4.1. Food Additives and Pesticide Residues

The issue of food safety has always been closely intertwined with human life, making the accurate detection of pesticide residues or food additives a significant concern for countries worldwide. Traditional methods of detecting pesticide residues rely on laboratory sampling and necessitate a series of operations, including sample pretreatment. For example, one commonly used method is high-performance liquid chromatography (HPLC), which is complex and time-consuming. Consequently, there is an urgent need for a rapid and simple method to detect food additives or pesticide residues. SERS is anticipated to be utilized for the on-site detection of pesticide residues and food additives due to its speed and simplicity. For instance, SERS substrates based on silver nanostructures have successfully detected malachite green additives (a cancer-causing agent) in apple juice and lake water, as well as mercury ions in river water, among others [71,72,73]. However, the preparation of SERS substrates faces some challenges, such as low sample collection efficiency and difficulty in achieving accurate detection. Therefore, the development of flexible SERS substrates is expected to significantly improve the sample collection efficiency and expand its application scope. For example, Zhong et al. [74] prepared a flexible SERS substrate with good flexibility, high transparency, and a strong SERS effect. By self-assembling gold nanoparticles in a polymethylmethacrylate flexible template, a gold nanoparticle/polymethylacrylate film SERS substrate was prepared and utilized to successfully detect pesticide residues in fish epidermis. Furthermore, Chen et al. [75] prepared a silver-coated carbon nanosphere core–shell structure as a SERS substrate and used it to successfully detect melamine in food.

In addition to the issue of excessive food additives and pesticide residues, there are also many harmful substances in the environment that are not detrimental to human health, such as organic substances and heavy metal ions. For example, waste from industrial production contains a large number of heavy metal ions, such as lead ions, chromium ions, and mercury ions, which pose a serious threat to human health and the ecological environment [76]. To address this issue, SERS technology is widely used for the in situ detection of pollutants in water bodies due to its advantages of high detection sensitivity, short detection time, low detection cost, and low susceptibility to water interference. For instance, the elemental valence of chromium has a great relationship with its toxicity, and Cr(VI) is the most toxic hexavalent chromium ion, which easily accumulates in the human body and causes various diseases. Generally, Cr(VI) exists in water as the compound CrO_4_^2−^, which is an important indicator of environmental pollution. Therefore, a method is needed to rapidly detect Cr(VI) in water. Zhao et al. [77] used TiO_2_ nanocolloids sensitized by alizarin red S to successfully detect low concentrations of Cr(VI) with a detection limit reaching 0.6 μM, which is lower than the maximum value of Cr(VI) in potable water stipulated by international regulations. Yu et al. [78] prepared a handheld silicon nano-heterostructure and used it for the simultaneous detection of Hg^2+^ and Pb^2+^ in waste. The substrate was based on silicon wafers covered with silver nanoparticles, which were also modified with 4-aminothiophenol molecules. The handheld SERS substrate combines sensitivity, selectivity, and convenience to rapidly identify Hg^2+^ and Pb^2+^ from ten other interfering metal particles, with detection limits of 9.9 × 10^−11^ M for Hg^2+^ and 8.4 × 10^−10^ M for Pb^2+^, which are two orders of magnitude lower than the U.S. Environmental Protection Agency emission standards, and can be used for the practical detection of Hg^2+^ and Pb^2+^ in industrial waste. Moreover, Hyanes et al. [79] prepared decanethiol monolayer-modified silver nanoparticles that were successfully used for the detection of anthracene and pyrene, as well as other lake pollutants. To meet the increasing demand for detection, more types of SERS substrates need to be developed for practical detection.

### 4.2. Biomedical Applications

A multitude of biosensors can be utilized to detect numerous biological samples and diseases, owing to their highly sensitive SERS effect. This encompasses a wide array of DNA, viruses, cancers, diabetes, neurological disorders, and cardiovascular diseases [34,80,81,82,83,84]. When analyzing the microenvironment of an organism, the detection of Raman signals is intimately linked to three parameters [85]: the nature of the substance undergoing analysis, the nature of the microenvironment within the organism, and the nature of the Raman-enhanced material. In modern medicine, the presence of certain specific molecules in an organism indicates the potential occurrence of pertinent disease. To achieve therapeutic objectives, the parameter levels of these specific molecules must be quantified, subsequent to which an accurate diagnosis can be formulated to administer appropriate treatment. Presently, while SERS technology can be successfully employed for the detection of degenerative diseases, such as the detection of amyloid-specific molecules for the diagnosis of Creutzfeldt–Jakob’s or Alzheimer’s disease, challenges persist in the detection of infectious diseases, such as viruses, bacteria, fungi, or other malignant tumors, which do not evidently contain specific molecules. Consequently, the development of more efficient SERS substrates and assays for the precise and sensitive identification of specific molecules associated with other diseases remains a formidable challenge.

**Figure 4:**
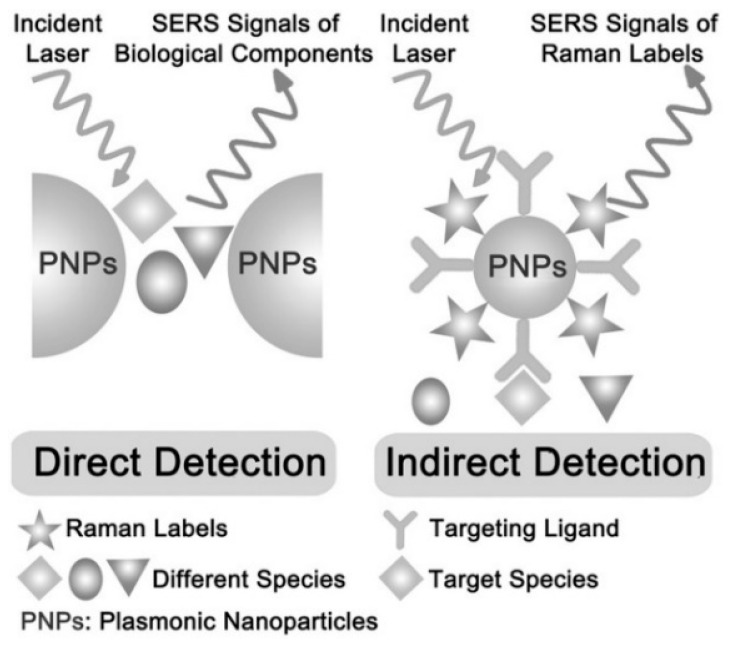
Schematic diagram of direct and indirect detection using SERS technology [86]. Copyright © 2015 WILEY-VCH Verlag GmbH & Co. KGaA, Weinheim.

The application of SERS detection technology in biomedicine is categorized into direct and indirect detection, as depicted in Figure 4 [86]. For biological macromolecules, direct detection is more prone to interference from the complex biological microenvironment, whereas indirect detection techniques can mitigate such interference. Indirect detection requires the preparation of SERS-labeled macromolecules, which are derived from distinct functional structures, generally comprising a plasmonic particle, a Raman probe molecule, and a protective shell with targeting properties. This SERS-labeled macromolecule with targeting properties can effectively circumvent the interference of other non-specific substances in the biological microenvironment and enhance the specificity of SERS detection.

Hepatitis B virus (HBV) is the causative agent of hepatitis B, a life-threatening disease. According to statistics, there are approximately 350 million HBV carriers worldwide, with 3000 deaths annually. HBV carriers are individuals with HBV who do not exhibit symptoms of hepatitis B and possess normal liver function. However, HBV carriers can still transmit the virus to healthy individuals. Consequently, there is an urgent need to develop a simple, rapid, sensitive, and reliable HBV test. Conventional HBV assays are based on the measurement of specific light absorbance, while existing assays such as the enzyme-linked immunosorbent assay (ELISA) and the electrochemical assay (ECA) often entail numerous complex intermediate steps, which are time-consuming and expensive. Subsequently, Adem et al. [87] prepared a SERS sensor for HBV detection. They attached the DNA molecular strand (labeled with indocyanine green) and the DNA capture strand to the surface of free gold nanoparticles via HBV DNA. As a result, they successfully distinguished and detected the HBV DNA target using the SERS sensor, with a minimum detection limit of 0.44 Μm. Furthermore, the detection limit of the sensor was further reduced due to the fact that gold nanoparticles are more prone to aggregation at approximately 37 °C than at 25 °C, thereby generating a stronger electromagnetic field. This study demonstrates that the ultra-high sensitivity of the SERS sensor holds great potential for the selective detection of other unlabeled viruses and proteins. Gorali et al. [88] successfully prepared a plasmonic nanopore in a thick nanoporous film, which was utilized to investigate, by means of SERS, the interaction between the metallic surface of the pore and a long-chain double-strand DNA molecule free to diffuse through the pore. Chourpa et al. [89] employed the SERS technique to study the effect of topoisomerase II inhibitors on corresponding cancer cells and found that topoisomerase II inhibitors potently inhibit tumor cell activity. Cao et al. [90] successfully detected immune response recognition using SERS and immunization techniques. Additionally, Zheng et al. [91] obtained the Raman spectrum of chloroferricyclohexine using the SERS technique. In conclusion, SERS offers advantages such as rapidity, simplicity, non-destructiveness, and sensitivity, and holds great promise for applications in biomedicine. It is noteworthy that when the SERS technique is applied in clinical testing, it is often necessary to consider the complex human environment, as serum and plasma contain valuable information for clinical diagnosis, and extensive data processing is typically required for analysis. However, the SERS substrate cannot function effectively in undiluted plasma. To address this challenge, Sun et al. [92] were the first to synthesize a quasi-3D gold nanostructure array and successfully used it for real-time monitoring of specific drug concentrations in undiluted human plasma. The main design principle involves a hierarchical surface modification of the gold nanostructures. Initially, a layer of self-assembled probe molecules is adsorbed onto the surface of the gold nanostructures, followed by the wrapping of a layer of amphiphilic polymer brushes to effectively prevent protein fouling. This SERS substrate exhibits a very fast response time and ultra-high sensitivity when used for target substance testing. Furthermore, the SERS substrate can be employed to monitor real-time concentrations of several other drugs present in undiluted plasma, such as antidepressants and two other anticonvulsants [93].

**Figure 5:**
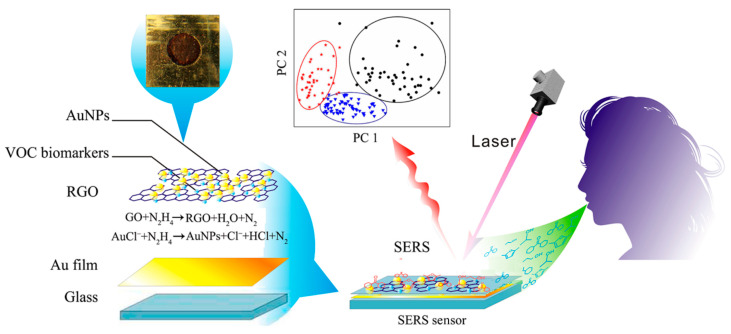
Schematic photographs of breath analysis based on a SERS sensor. The red five-pointed star, blue triangle and black spot are real breath samples of advanced gastric cancer patients, early gastric cancer patients and healthy persons, respectively [98]. Copyright © 2016 American Chemical Society.

Cancer represents one of the most common groups of malignant tumors and is a highly lethal disease. Governments are committed to implementing various measures to prevent cancer-related deaths, with early preventive diagnosis being one of them. Studies have shown that the survival rate of patients diagnosed with cancer at an early stage is higher than that of patients diagnosed at an advanced stage [94,95]. SERS tags are used to capture specific metabolites in serum and analyze their composition for early cancer detection. Different specific metabolites correspond to different types of cancer and the stage of cancer development. For example, synthesized sea urchin-like gold nanoclusters have been successfully used to detect mutated genes in the epidermal growth factor [96] and the analysis of specific substances in exhaled breath has been used to diagnose different stages of gastric cancer [97]. Similarly, Cui et al. [98], used a SERS sensor designed to analyze saliva for the diagnosis and differentiation of early or advanced gastric cancer. As shown in Figure 5, the SERS spectra of bio-specific substances in four different sets of saliva samples are presented: blank, control, advanced cancer patient, and early cancer patient. By analyzing bio-specific substances in saliva, it is possible to diagnose patients at different cancer stages. In conclusion, this technique holds great promise for clinical applications.

**Figure 6:**
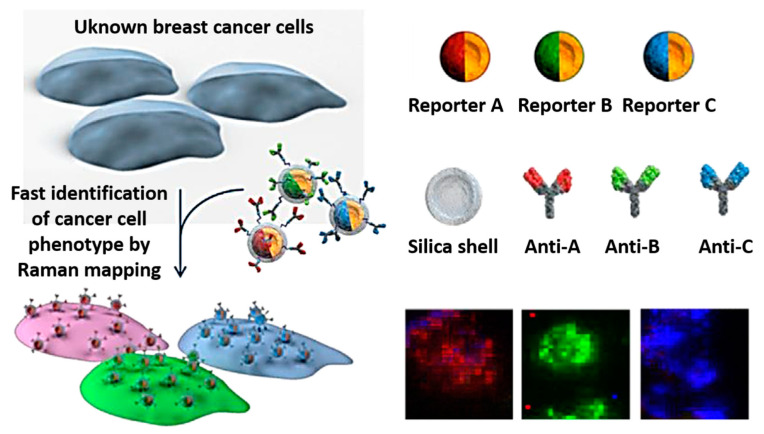
Rapid detection and quantitative evaluation of phenotypic markers on cell surface membranes using the SERS imaging technique [100]. Copyright © 2013 Elsevier B.V.

Nanomaterial-based SERS technology can also be utilized for cellular imaging and intracellular drug detection. Compared with traditional fluorescent probes, Raman imaging exhibits higher sensitivity, better recognition ability, and enhanced anti-interference properties, which have contributed to the advancement of SERS in cellular imaging applications. In 2008, Nie et al. [99], were the first to apply the SERS imaging technique to in vivo tumor detection. Since then, SERS imaging has frequently been used in tumor imaging research and applications. In vivo tumor detection typically involves the delivery of SERS probes into the organism and waiting for these probes to accumulate in the tumor tissue before imaging the tumor in vivo. For example, hollow gold nanoparticles can be used to detect marker-specific molecules of the human epidermal growth factor causing cancer, while SiO_2_-encapsulated hollow gold nanoparticles are capable of detecting marker-specific molecules of breast cancer cells and imaging breast cancer cells. As depicted in Figure 6, cellular imaging can be significantly enhanced by detecting specific molecules of cancer cells. Furthermore, after the cells have absorbed these specific molecules, the detection of these molecules can be utilized to ascertain the location of the cells and obtain the corresponding SERS images [100]. Additionally, Ren et al. [92] also used 4-mercaptopyridine to modify the gold nanoparticles, and subsequently employed bovine serum albumin to protect the entire system, thereby preparing SERS probe molecules and successfully monitoring the intracellular pH distribution and distribution map.

### 4.3. Reaction Process Monitoring, Sensing, and Imaging

**Figure 7:**
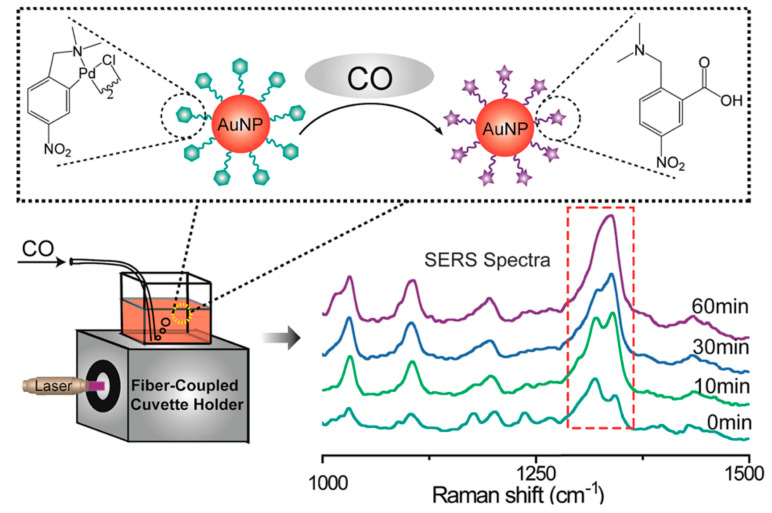
Schematic illustration of the in situ monitoring of palladacycle-mediated carbonylation with SERS. The peaks at the red dashed frame may be assigned to the stretching vibration of C-N, C-H, and C-Pd bond [85]. Copyright © 2015 Royal Society of Chemistry.

Compared with traditional sensing techniques, SERS technology possesses the capability for non-destructive and real-time in situ monitoring. As such, it can be utilized to monitor transient intermediates generated in chemical reactions related to non-homogeneous catalysis and to analyze the reaction mechanism, providing an important foundation for the optimization of the reaction system. Consequently, SERS technology is frequently employed to investigate the process of catalytic reactions. For example, Ding et al. [101] prepared a three-dimensional Fe_3_O_4_@Au@Ag nanoflower-like structure assembled with a nano-chain SERS substrate via in situ reduction and magnetic field-induced assembly. This substrate exhibits high detection sensitivity and signal reproducibility and has been successfully used for non-destructive real-time monitoring of the 4-nitrophenol reduction reaction. As shown in Figure 7, Hu et al. [85] prepared a reaction-mediated SERS nano-substrate consisting of newly synthesized gold nanoparticles modified with p-cyclic palladium complexes. This substrate can be used for the in situ monitoring of the p-cyclic palladium complex-mediated carbonylation process in a simple and convenient manner, without cumbersome pre-treatment. Furthermore, the kinetic parameters of the reaction can be deduced from the relationship between the reaction time and the ratio of SERS signal intensity at 1319 cm^−1^ and 1338 cm^−1^. Moreover, Ouyang et al. [102] prepared a SERS substrate based on Ag nanoparticle-modified β-cyclodextrin Pickering emulsion, which exhibits high SERS activity and is capable of detecting the generation of target compounds as well as monitoring the reaction process. This substrate can also be used for the real-time monitoring of the oxidation of o-phenylenediamine for the preparation of 2,3-diaminophenolazine.

In addition to monitoring reactions, SERS techniques are also effective in sensing and imaging. For example, Xia et al. developed a super-long silver nanowire-based SERS detection strategy for simultaneous sensing. A surface-roughened silver nanowire with a high aspect ratio is prepared at a nanoelectrode tip using a Cu-mediated oxidation process and is then modified by pH-sensitive 4-mercaptobenzoic acid to form a silver nanowire pH sensing probe [103]. As for SERS imaging, the Luis group has prepared a well-defined Raman fingerprint for SERS imaging. Their studies have demonstrated the incorporation of plasmonic nanoparticles within 3D scaffolds for SERS-based sensing and imaging of model molecules and cells, respectively. While bare ligand-free anisotropic plasmonic nanoparticles enable label-free sensing of cell-secreted metabolites, nanoparticles labeled with Raman-active molecules on their surface, known as SERS tags, have also been developed [104,105,106,107].

### 4.4. Archaeology and Art

**Figure 8:**
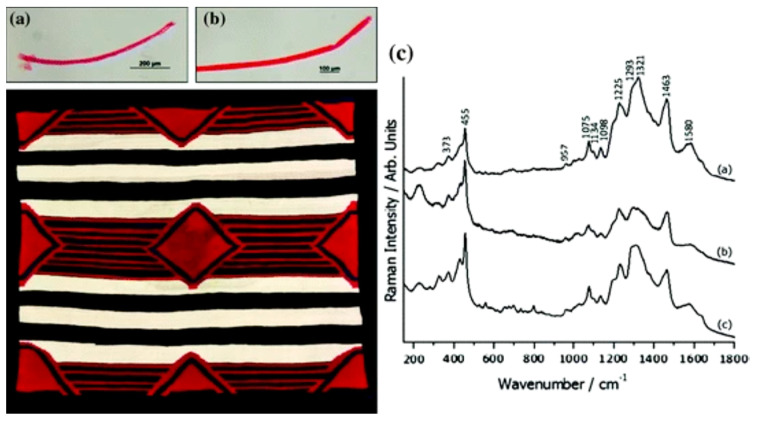
The SERS spectra of two red fibers removed from the blanket (**a**,**b**) are in accordance with that obtained from a reference wool sample dyed with cochineal (**c**) [110] Copyright © 2016, Atlantis Press and the author(s).

The SERS technique has been used in the field of cultural and artistic heritage for over thirty years, owing to its exceptional molecular selectivity, ultra-high sensitivity, and non-destructive sample detection capabilities. It is particularly adept at detecting and identifying organic colorants, such as various natural dyes and certain synthetic dyes. Due to its unique characteristics compared to traditional testing methods, the SERS technique has facilitated the identification of numerous types of organic colorants from archaeologically and artistically significant samples. Over the past decade, researchers have employed SERS technology to record the characteristic spectra of different dyes and have gradually established a comprehensive database of dye “fingerprints”. Investigating the sources of dyes used in the creation of historical artifacts and their potential chemical behaviors during the dyeing process can assist archaeological researchers in further unraveling the mysteries of these artifacts. Understanding the original shape and color of the artifacts can also enable researchers to gain a more comprehensive and profound insight into the artist’s actual intentions, as well as facilitate better discrimination between forged historical artifacts and authentic works of art. Figure 8 depicts a Navajo blanket from the Art Institute of Chicago. The SERS spectra of two red fibers in the Navajo blanket were obtained using the SERS technique [108]. Upon comparative analysis, the spectra were found to be consistent with those of a wool product stained with rouge. In addition to the natural dye, carbamic acid, detected in the blanket, two organic colorants, cochineal and β-naphthol dye, were detected using a 785 nm laser. This provides an important reference for accurately deducing the actual time of preparation of the Navajo blankets. Furthermore, Chen et al. [109], obtained SERS spectra of certain acids from small pieces of filter paper stained with reference dye solutions and coated with a thickness-controlled layer of silver nanoparticles. This paved the way for the direct identification of organic colorants on microscopic fragments from art objects. Additionally, Jurasekova et al. [110], described the utilization of photo-reduced silver nanoparticles to generate SERS signals from reference dyed fibers. The application of this method was initially demonstrated as a proof of concept for wool and silk samples dyed in the laboratory with flavonoid-containing weld dye [111].

The SERS technique, currently applied in the field of archaeology and art, still faces some challenges, such as the complexity of artifacts and artworks. During SERS detection, various chemicals are adsorbed onto the metal SERS substrate, seriously interfering with the detection of the target dyes. For artifacts with mixed dyes, the metal SERS substrate can often simultaneously obtain the combined spectra of different dyes, and the combined spectral peaks are complex and irregular. The spectra of two dyes with a disparity in detection limits may also exhibit a covering relationship. Therefore, it is very difficult to fully resolve the colorants in the SERS spectra of mixed colorants, and the development of a selective SERS substrate may solve this problem.

## 5. Conclusions and Prospects

Herein, a powerful analytical technique named SERS, distinguished by its high sensitivity and specificity, is introduced. Since its discovery, SERS has been widely applied across various fields, including physics, chemistry, and analysis, encompassing diverse aspects such as materials science, environmental science, biology, and medicine.

To obtain SERS substrates with a high sensitivity, efficiency, and repeatability, it is often necessary to design the preparation of nanostructures with numerous “hot spots” to achieve a more accurate, rapid, and reproducible SERS detection. In addition, statistical data analysis is typically required to achieve the quantification and identification of multiple components in complex mixtures, which is a crucial condition for the widespread application of SERS substrates. Furthermore, theoretical modeling and experimental validation are essential to further enhance the effectiveness of SERS applications in various scientific and industrial fields.

Over the past decades, SERS has been extensively utilized in the field of sensing. However, the progress of SERS sensing for practical applications has been hindered by the stability of existing instruments and SERS substrates. To overcome these challenges, future consideration could be given to developing more refined SERS probes to address the issue of uneven SERS substrate signals. For instance, loading a single layer of silver nanoparticles onto the surface of a nanoscale probe could enable nanoscale SERS detection. With the extraordinary technological advancements in the development of related instruments and the rapid progression of nanoscience and technology, the preparation technology of SERS substrates and the degree of control over substrate structures are becoming increasingly mature. It is anticipated that research outcomes related to SERS will experience explosive growth and further promote the development of SERS in numerous diverse directions.
